# Proton Migration-Modulated n‑Doped Poly(benzodifurandione) Organic Electrochemical Transistors Used for Neuromorphic Computing Applications

**DOI:** 10.1021/acsenergylett.5c02076

**Published:** 2025-10-02

**Authors:** Ignacio Sanjuán, David Franco, Qun-Gao Chen, Chu-Chen Chueh, Wen-Ya Lee, Antonio Guerrero

**Affiliations:** † Institute of Advanced Materials (INAM), 16748 Universitat Jaume I, 12006 Castelló, Spain; ‡ Department of Chemical Engineering and Biotechnology, and High-Value Biomaterials Research and Commercialization Center, National Taipei University of Technology, Taipei 106344, Taiwan; § Department of Chemical Engineering, 34877 National Taiwan University, Taipei 10617, Taiwan

## Abstract

Neuromorphic computing has emerged as a promising technology that can overcome the limitations that traditional systems face in the Big Data era. Organic electrochemical transistors (OECTs) are potential candidates for artificial synapses in neuromorphic hardware. However, due to their ambient instability, n-type OECTs have not been successfully applied to date in organic artificial synapses, limiting the fabrication of complementary logic circuits. In this work, we prove the potential of the n-doped poly­[benzodifurandione] (referred to in the literature as PBFDO or n-PBDF) polymer to fabricate high-performance n-type OECTs for neuromorphic applications using protons as the principal migrating ions. We demonstrate that n-PBDF-based OECTs show high stability and dual working modes (accumulation and depletion) in a NaPF_6_ electrolyte. The devices exhibit resistive switching and synaptic plasticity promoted by the H^+^ of the electrolyte. The n-PBDF OECTs also show high-quality long-term potentiation (LTP)/depression (LTD) behavior at low gate voltages (0.8 V) and short pulses (50–500 ms). The applicability of n-PBDF OECTs in neuromorphic computing is successfully validated by simulation with a deep neural network (DNN) model for handwritten digit recognition with different Gaussian noise levels. This work opens new avenues for the future development of n-type OECTs for building (bio)­electronic circuits, such as (bio)­sensing and neuromorphic computing.

## Graphical abstract

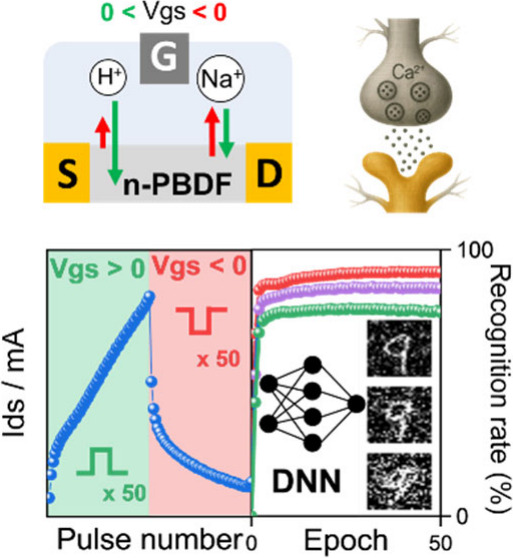

Silicon-based chips have traditionally been used in electronics because of their reliability. Nevertheless, from an energy consumption perspective, this technology is inefficient and limited by the von Neumann architectures, especially in modern applications requiring data-heavy processing, such as artificial intelligence (AI), big data, and edge-computing, as well as tasks requiring parallel processing or adaptivity.^1^ Neuromorphic computing, as an emerging technology, overcomes these limitations by simulating the dynamic behavior of synapses and neurons in the human brain.^2^ This new paradigm thus offers large-scale parallel and event-driven data processing, low energy consumption, and adaptivity achieved by learning from data.

Organic electrochemical transistors (OECTs) are promising electronic platforms for neuromorphic computing as their operating principle is analogous to that of biological synapses.^3–5^ In the biologic memory, a spike propagates along the axon of a neuron and is transmitted to another neuron via a synapse. This transmission is mediated by chemical signals known as neurotransmitters and is highly energy-efficient. Alternatively, OECTs work by placing organic materials in a channel between two electrodes and manipulating their conductivity through volumetric doping and dedoping. Ions penetrating or releasing from the organic material alter the conductance of the channel.^6,7^ This mechanism resembles the ion- and neurotransmitter-induced signal transmission processes observed in biological synapses. The key component of OECTs is the channel layer material, which must have the ability to couple ionic and electronic transport, respond dynamically to ionic fluxes,^8,9^ and show synaptic plasticity.^10^ Tailorable organic mixed ionic–electronic conductors (OMIECs) are ideal candidates to meet these requirements.^11^ Although significant progress has been made using p-type OMIECs such as poly­(3,4-ethylenedioxythiophene):poly­(styrenesulfonate) (PEDOT:PSS),^12,13^ the development of high-performance n-type OMIECs that are stable in air and water (electrolyte) remains challenging, and their applications in artificial synapses have been rarely reported to date.^14–17^

The development of n-type OMIECs will enable the development of complementary circuits when paired with p-type OMIECs, thereby achieving lower power consumption, higher resilience to noise, and better signal stability in both logic circuits and neuromorphic circuits.^18^ Recent advances in n-doped conjugated polymers have opened up new opportunities in this field. Tang et al. reported a facile synthesis method for highly conductive n-doped poly­(benzodifurandione) (PBFDO or n-PBDF), which exhibits outstanding electrical conductivity, high doping level, and good stability under ambient conditions.^19–21^ In the last three years, several studies have explored the outstanding properties of n-PBDF thin films in transparent electrodes,^22,23^ electrophysiological monitoring,^24^ thermoelectric textiles,^25^ and OECTs,^26,27^ confirming the potential of this n-type polymer for organic electronics. Nevertheless, despite their potential, characterization studies on n-PBDF OECTs for neuromorphic functionalities remain limited.

In this work, we demonstrate the potential application of n-PBDF in OECTs for neuromorphic computing. We conducted a comprehensive electrochemical characterization of the OECTs based on the n-PBDF thin films deposited on an ITO-patterned substrate, with a particular focus on special features crucial for neuromorphic computing. The results indicate that n-PBDF-based OECTs can mimic fundamental synaptic behaviors, including dynamic current modulation and plasticity-like responses such as spike-width-, spike-voltage-, and spike-frequency-dependent plasticity. The memory response highly depends on the pH of the electrolyte solution and it seems to be triggered by proton (H^+^) transport across the active channel. The performance of n-PBDF OECTs in practical applications was validated by simulating a deep neural network (DNN), which achieved high accuracy (>96%) in recognizing handwritten digits through weight modulation based on n-PBDF OECT behavior. The reliability of the device was further demonstrated by adding Gaussian noise during DNN training to simulate real-world noise environments, with results showing good performance.

PBFDO or n-PBDF was synthesized following the reported procedure and described in the Experimental Section.^22^ The synthesis employed CuAc_2_ as a catalyst instead of tetramethyl-1,4-benzoquinone (TMQ), yielding a partially n-doped polymer (Figure 1a), with protons serving as counterions. The full conversion of the monomer (benzo­[1,2-*b*-4,5-b0]­difuran-2,6-(3H,7H)-dione) BDF into the PBFDO polymer was confirmed by ^1^H NMR (Figure S1). The disappearance of the peaks located at 7.25 and 3.96 ppm of the spectrum, which correspond to the aromatic hydrogen and the methylene hydrogen of BDF, respectively, indicates its 100% conversion.

**Figure 1:**
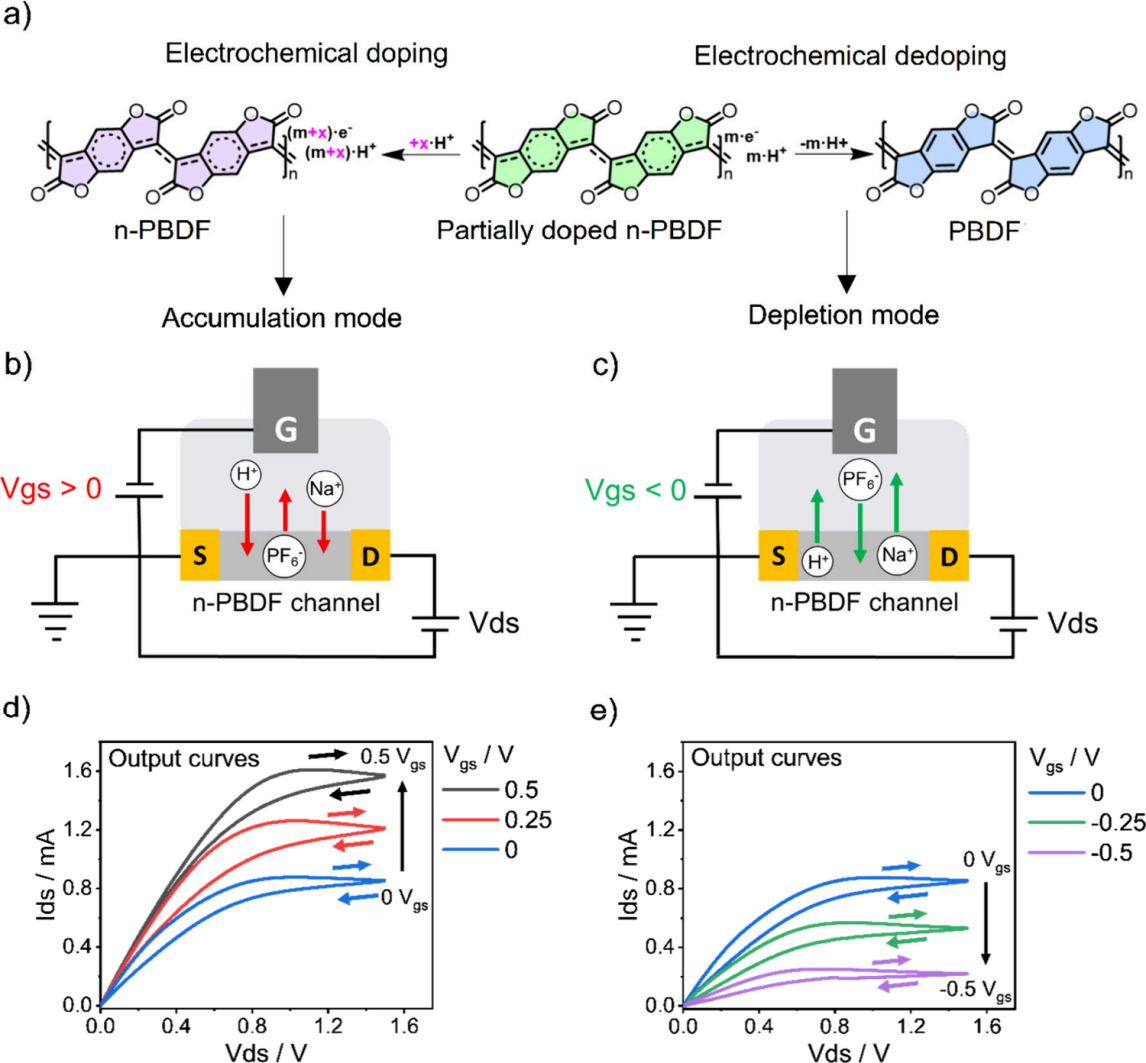
(a) Scheme depicting the doping and dedoping processes of the polymer n-PBDF with its chemical structure changes. Schematic illustration of ion movement (b) under positive gate bias and (c) under negative gate bias. Output characteristic curves of an OECT fabricated using partially doped n-PBDF working in (d) accumulation mode and (e) depletion mode. Electrolyte: 0.1 M NaPF_6_. Gate: Ag/AgCl wire.

The electrical conductivity of n-PBDF was measured using the 4-probe method, combined with voltammetry and electrochemical impedance spectroscopy (EIS) techniques. The results showed that the electrical conductivity of the as-prepared polymer under dry conditions is 70 S cm^–1^ (Figure S2), which is quite high for n-type conductive polymers. Additionally, this conductivity is consistent with the results obtained by Ke et al. prepared under the same conditions in the absence of TMQ.

We fabricated the OECTs by depositing n-PBDF thin films on ITO-patterned glass substrates as described in the Experimental Section. The first step was to evaluate their performance in a transistor configuration using electrochemical characterization, both in accumulation mode and depletion mode, as will be shown below. The characterization was based on recording the *I–V* responses by varying the voltage between the gate and drain electrodes relative to that of the source electrode (Figure 1b,c). The gate and drain electrodes shared the same source reference potential, which was grounded to ensure stability. Herein, we used 0.1 M NaPF_6_ (pH ∼ 3) as the electrolyte for characterization to enable a stable response of the polymer according to the findings reported by Wu et al.,^24^ who demonstrated that electrolytes containing large anions (e.g., PF_6_^–^ or BF_4_^–^) enhance the operational stability of n-PBDF OECTs during dedoping process compared to those with small halide anions (e.g., Cl^–^ and Br^–^). Here, we confirmed that n-PBDF showed significant progressive degradation in the electrochemical response with increasing experimental cycles when 0.1 M NaCl (pH ∼ 7) was used as the electrolyte (Figure S3). Figure 1 summarizes the electrochemical properties of n-PBDF OECTs tested in the 0.1 M NaPF_6_ electrolyte. The results revealed that the synthesized polymer was partially doped (Figure 1a). As a result, the fabricated n-PBDF OECTs can exist in either a doped or dedoped state (Figure 1b,c), allowing the device to operate in either accumulation mode or depletion mode depending on the magnitude and polarity of the gate-source voltages (*V*_gs_). A positive bias of the gate relative to the source induces the insertion of positive ions (e.g., H^+^ and Na^+^) into the polymer structure (Figure 1b), thereby stabilizing electron accumulation (accumulation mode). Conversely, a negative bias of the gate relative to the source causes the withdrawal of H^+^/Na^+^ (Figure 1c), positive ions that stabilize the mobile electrons in the polymer structure, reduces the load charge carriers, and thus lowers the electrical conductivity (depletion mode). For the sake of simplicity, we refer to H^+^/Na^+^ movement due to the small size of the cations in comparison to the large anion PF_6_^–^. However, it is also expected that this anion can enter and exit the polymer under appropriate conditions. We show below that H^+^ plays a major role in potentiation experiments.

Regarding electrical measurements, Figure 1d shows the output curves under drain-source voltage (*V*_ds_) ranging from 0 to 1.5 V (30 mV s^–1^) and various positive *V*_gs_. As *V*_gs_ increases, the current flowing through the channel (*I*_ds_) increases, indicating a progressive doping in the n-PBDF within the channel, consistent with a gate voltage-induced ion insertion phenomenon. The *I–V* curves show capacitive hysteresis and a plateau at *V*_ds_ ≈ 0.8 V, where the maximum current is observed. When *V*_gs_ is set to 0.5 V, the current on the plateau is approximately twice that at *V*_gs_ = 0 V. Additionally, Figure 1e shows the output curves during channel material dedoping under the same *V*_ds_ window (30 mV s^–1^) and different negative *V*_gs_ values. In this case, the *I*_ds_ current decreases as *V*_gs_ becomes more negative, indicating that the n-PBDF channel is being dedoped. At *V*_gs_ = −0.5 V, the *I*_ds_ current at the plateau of the *I–V* curve is four times lower than that at *V*_gs_ = 0 V.

The above results highlight the significant advantages of n-PBDF in the field of OECT applications, which are primarily attributed to its unique redox capabilities. This is also the first demonstration of n-PBDF’s dual working mode in OECTs, as well as the first example of an n-type OECT capable of operating in both accumulation and depletion modes. Additionally, we demonstrate that the system is highly sensitive to small changes in the gate voltage *V*_gs_, indicating that if the introduction/release of ions from the organic material can serve as the fundamental physical/chemical process for memory, the memory can operate at very low voltages, thereby enabling energy-efficient systems.

The as-prepared n-PBDF OECTs operate in both accumulation and depletion modes, suggesting that these devices can accurately simulate neural processes such as synaptic excitation and inhibition or promotion/forgetting behaviors by increasing or decreasing conductance. This is essential for constructing analog, trainable circuits. Therefore, we investigated the performance of these devices in memory and neuromorphic computing.

Figure 2a presents the characteristic transfer curves of the OECT, obtained by cycling *V*_gs_ between −0.5 and 0.5 V at a rate of 30 mV s^–1^, followed by increasing *V*_ds_. The *I*_ds_ current in the *I–V* curves is lower at negative *V*_gs_ values and increases with the positive variation of *V*_gs_, evidencing that the n-PBDF OECT has the potential to operate in a dual-mode (accumulation/depletion) regime. The transfer curve registered in the saturation regime (*V*_ds_ = 1.2 V) revealed that the n-PBDF OECTs shows a moderate normalized transconductance (*g*_m_^norm^) of 0.124 S cm^–1^ (Figure S4). The transfer curves show inductive hysteresis in all cases,^28,29^ with the hysteresis magnitude increasing with *V*_ds_. In general, at *V*_gs_ ≈ 100 mV, the OECT enters a high-conductive state (ON state), and during the backward scan process, the device returns to a low-conductance state (OFF state) when *V*_gs_ drops to a sufficiently low value of ∼−500 mV. This resistive switching behavior proves the potential of the n-PBDF OECT for memory and neuromorphic applications. Therefore, we investigated the response of the OECT to gate voltage (*V*_gs_) pulses to emulate processes occurring within a neural network. Figure 2b shows the typical spike-width-dependent (SWD) plasticity exhibited by the OECT, where a positive *V*_gs_ pulse (0.8 V) causes an increase in *I*_ds_, with the extent of the increase depending on the pulse width. The longer the pulse duration, the higher the measured *I*_ds_, as more cations are inserted into the n-PBDF channel. Once the transition ceases, *I*_ds_ returns to its resting state, but not immediately; instead, it decays at a rate proportional to the duration of the applied *V*_gs_. The longer the pulse duration, the slower the decay of the transient current, as the cations inserted into the polymer structure cannot quickly return to their initial state.

**Figure 2:**
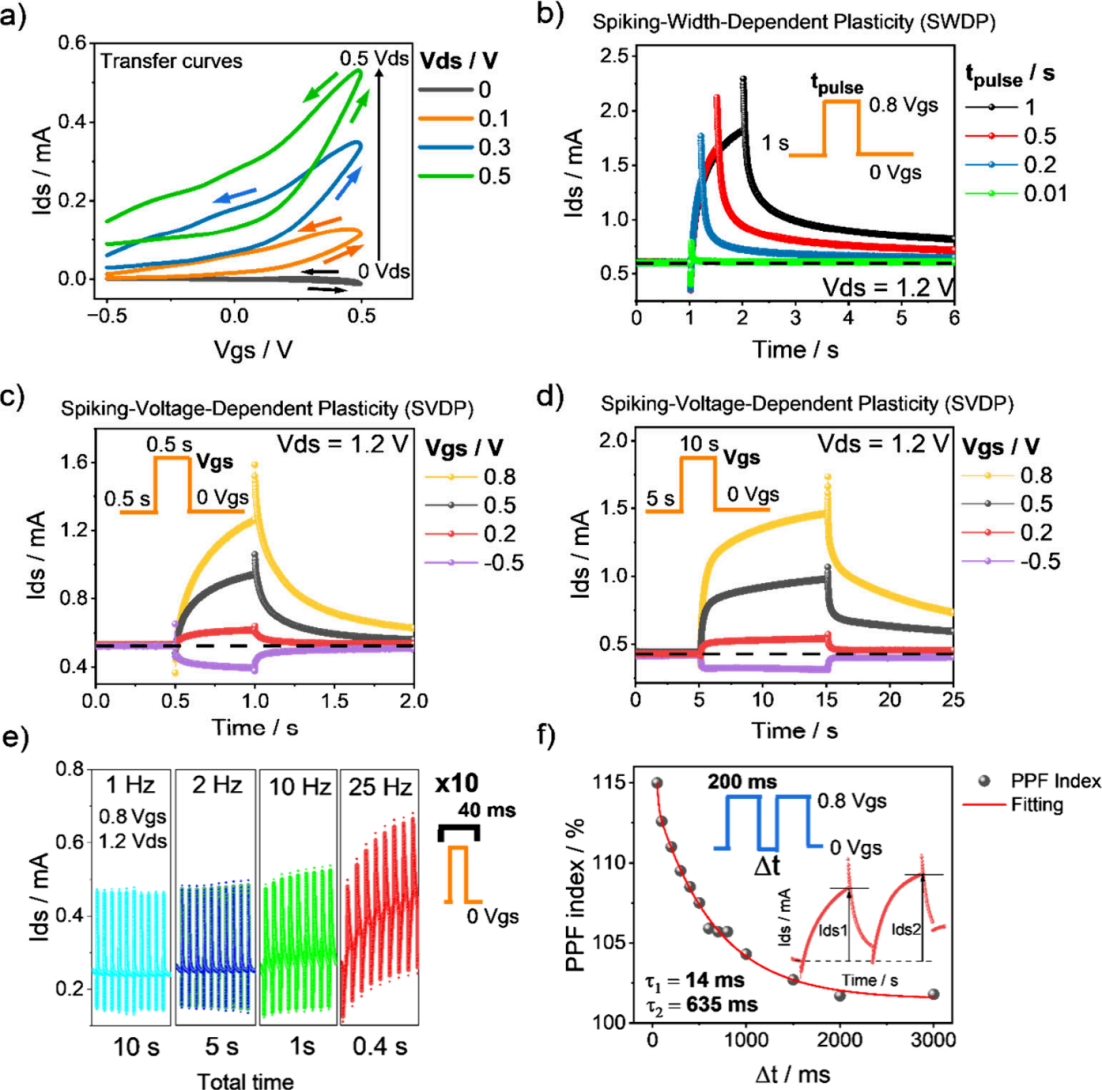
(a) Transfer characteristic curves of the n-PBDF OECT under different drain-source voltages (*V*_ds_). (b) Spike-width-dependent plasticity (SWDP) of the device under *V*_gs_ = 0.8 V pulses with different durations. Spike-voltage-dependent plasticity (SVDP) of the device for (c) 500 ms and (d) 10 s pulses of different *V*_gs_. (e) *I*_ds_ response of the devices under short pulse trains at different frequencies. (f) Paired-pulse facilitation index and fitted values calculated by measuring the current ratio between two consecutive pulses with different separation times.

Figure 2c,d also confirms the spike-voltage-dependent (SVD) behavior of the OECT. Higher *V*_gs_ pulses result in larger *I*_ds_ currents and a slower decay back to the initial OFF state. The curve of *I*_ds_ versus time in Figure 2d (obtained under long *V*_gs_ pulses) reveals that *I*_ds_ continues to increase throughout the pulse duration. Applying high *V*_gs_ (>0.5 V) and long pulse durations (>1 s) keeps the OECT in the ON state for a longer time, resulting in increased long-term conductance. For instance, applying 10 s *V*_gs_ pulses of 0.5 and 0.8 V results in a reading current (at reading *V*_gs_ = 0) that is at least 80 μA higher than the OFF state and persists for at least 40 s after the pulse (Figure S5). These observations suggest that the n-PBDF OECTs exhibit flexible tunability from volatile to nonvolatile behavior. Moreover, negative *V*_gs_ pulses induce the opposite behavior; that is, *I*_ds_ decreases relative to the initial state. This originates from the dual behavior of partially doped n-PBDF and can be utilized to emulate the depression or forgetting processes of neural networks.

To further assess the device’s response to synaptic-like signals, we applied trains of 10 consecutive pulses with different frequencies to the n-PBDF OECT. Figure 2e presents the time-dependent evolution of *I*_ds_ in the n-PBDF OECT under different pulse trains at different frequencies. The reading of *V*_gs_ was 0 V in all cases. The results show that *I*_ds_ increases during the reading step only when the frequency exceeds 10 Hz, evidencing that the pulse width and voltage are not the sole critical parameters and that the temporal interval between excitatory pulses also influences the potentiation process. Next, we investigated the paired-pulse facilitation (PPF) of the OECT by applying two successive *V*_gs_ pulses with different interval times (*Δt*). Subsequently, we calculated the PPF index, defined as the ratio of *I*_ds_ measured during the second pulse (*I*_ds_2) to *I*_ds_ of the first pulse (*I*_ds_1), as follows:

1 $$\mathrm{PPF index}\;\left(\%\right)=\frac{I_{\mathrm{ds}}2}{I_{\mathrm{ds}}1}\cdot 100$$

Figure 2e shows the relationship between the PPF index and *Δt*. The PPF index values were obtained from the corresponding *I*_ds_–*t* curves (Figure S6). For the n-PBDF OECT, the PPF index was 115% at *Δt* = 50 ms, followed by an exponential decay, reaching a steady-state value of ∼101% at *Δt* > 2 s. This curve can be fitted using a double-exponential decay function described in eq 2:^30^

2 $$\mathrm{PPF index}\;\left(\%\right)=C_{0}+C_{1}\cdot \exp\left(-\Delta t/\tau_{1}\right)+C_{2}\cdot \exp\left(-\Delta t/\tau_{2}\right)$$

where *C*_0_ represents the PPF index value to which the curve converges (101%); *C*_1_ and *C*_2_ are the weights of the slow and fast paired-pulse facilitation phases (*C*_1_ = 55% and *C*_2_ = 13%), respectively; and *τ*_1_ and *τ*_2_ are the characteristic relaxation times of fast and slow processes, respectively. The fitting of the experimental results with eq 2 leads to the following relaxation times: *τ*_1_ = 14 ms and *τ*_2_ = 641 ms. These values are in the same range as those reported for other n-type OMIEC-based OECTs.^16^ These relaxation times are biologically meaningful^15^ and demonstrate the short-term synaptic plasticity (STP) of the n-PBDF OECT. When a positive *V*_gs_ is applied, cations (H^+^ and Na^+^) are injected into the polymer channel, increasing the conductance of n-PBDF, thereby acting in a manner similar to neurotransmitters in biological synapses. When a second pulse is applied, if the speed is sufficiently fast, more cations are inserted before moving ions from the previous pulse returns to equilibrium, leading to their accumulation and thus potentiation.

In order to gain a deeper understanding of the role of the cation/anion electrolyte–polymer interactions in the changes in conductance caused by *V*_gs_ bias, we performed experiments using different electrolytes to compare their responses. First, to unravel the role of the anion during the doping process, we compared the electrolyte used in this work, 0.1 M NaPF_6_ (pH 3.5), to 0.1 M NaCl (pH 7). Alternatively, protons (H^+^) in the electrolyte at a given pH value may also be the primary factor in the memory response, leading to insertion into the polymer. For this reason, we also tested the OECT in combination with 0.1 M NaCl at pH 3.5. Figure 3a shows the change in the *I*_ds_ current of the n-PBDF OECTs over time when a positive *V*_gs_ = 0.5 V is applied in the different electrolytes. Before the experiment, the devices were reset to the OFF state by applying a negative *V*_gs_ = −0.5 V (I). The results show that all the devices undergo a similar rise in *I*_ds_ current of about 450 μA when the *V*_gs_ pulse starts, and reach a steady-state current during the pulse duration (II). The profiles of the *I*_ds_–time curves during the pulse are not significantly different among the different electrolytes, suggesting that anions have no significant influence on this process. Once the pulse is finished (III), the *I*_ds_ decays in all cases, but only the current in the device tested in 0.1 M NaCl (pH 7) returns fast to the same level as before the *V*_gs_ pulse was applied. In contrast, the experiments performed in 0.1 M NaPF_6_ (pH 3.5) and 0.1 M NaCl (pH 3.5) presents different behavior, as their *I*_ds_ currents after the decay post-*V*_gs_ pulse remains significantly higher (∼70 μA) than those in their initial state, revealing a long-term increase in conductance caused by the positive *V*_gs_ pulse. This indicates that H^+^ plays a key role in the change of conductance of n-PBDF OECTs, thereby affecting their synaptic plasticity, especially when a positive *V*_gs_ bias is applied. During the positive *V*_gs_ pulse, both H^+^ and Na^+^ are inserted into the polymer structure, but H^+^ must be held longer than Na^+^ within the polymer after the *V*_gs_ bias due to (electro)­chemical interactions (Figure 3b). To confirm the doping state of the n-PBDF OECTs after the positive *V*_gs_ pulse, we measured *ex situ* the UV–vis–NIR spectrum in the 350–1600 nm range of the n-PBDF OECT in different conditions: (1) as-prepared, (2) doped after the application of a 2 min long *V*_gs_ = +0.5 V pulse for doping, and (3) undoped after applying a 2 min long *V*_gs_ = −0.5 V pulse for dedoping in 0.1 M NaPF_6_ (pH 3.5). Figure 3c shows the UV–vis–NIR spectrum of the doped n-PBDF OECT compared to that of the as-prepared device. The spectrum after doping at *V*_gs_ = +0.5 V shows higher absorption than the as-prepared n-PBDF OECT over the whole range measured. The higher absorption in the range of 1000–1600 nm, assigned to the polaron–bipolaron absorption band, confirms the higher doping that remained in the n-PBDF film after the *V*_gs_ pulse. Subsequently, when the n-PBDF OECT is dedoped for 2 min at a *V*_gs_ pulse of −0.5 V (Figure 3d), the absorption is decreased in the whole window, especially in the polaron–bipolaron absorption region due to the dedoping process. The different postpulse decay profile observed between the experiment with NaPF_6_ (pH 3.5) and NaCl (pH 3.5) suggests that anions may influence the withdrawal of cations from the channel to the electrolyte since both electrolytes have the same amount of H^+^ and Na^+^ but different anions.

**Figure 3:**
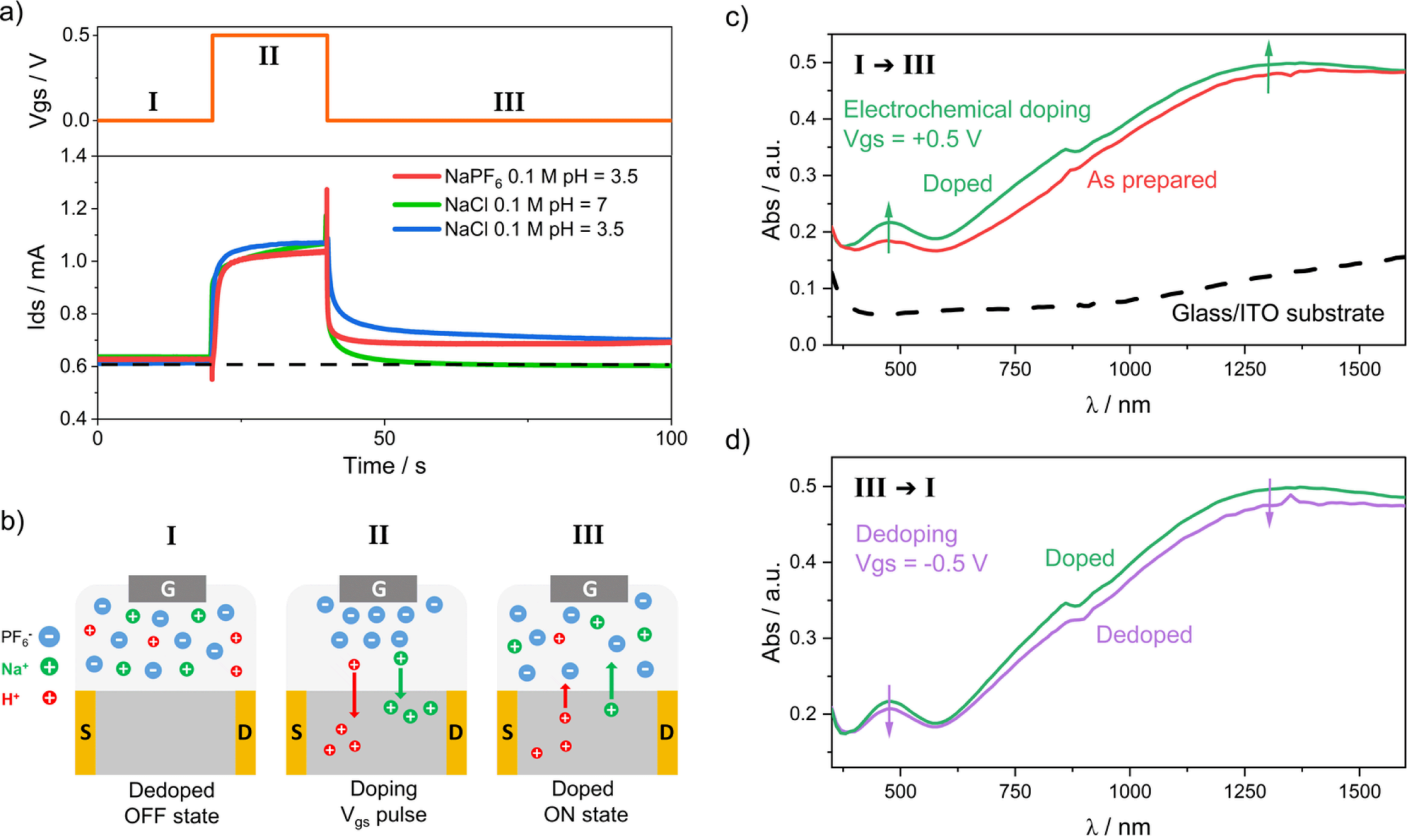
(a) *I*_ds_ current–time response under a gate voltage *V*_gs_ pulse for the n-PBDF OECTs with different electrolytes. The applied *V*_gs_ program is described at the top. The OECTs were previously deactivated to the OFF state by applying a negative *V*_gs_ pulse of −0.5 V (20 s). (b) Schematic description of the electrolyte–polymer interaction for the doping process derived from the application of the *V*_gs_ pulse. (c) UV–vis–NIR spectra measured for as-prepared (red) and doped (green) n-PBDF OECTs. Doping was carried out at *V*_gs_ = +0.5 V for 2 min in 0.1 M NaPF_6_. The spectrum of the glass/ITO substrate is included as reference. (d) UV–vis–NIR spectra measured for the doped n-PBDF OECT (green) and a dedoped n-PBDF OECT (violet) after 2 min at *V*_gs_ = +0.5 V in 0.1 M NaPF_6_.

Different anions are also proved to have a key role in the dedoping process of the n-PBDF OECTs, as the *I*_ds_-time curve observed for the NaPF_6_ electrolyte is different from those registered for the NaCl-based electrolytes at pH 7 and 3.5 (Figure S7), which in turn are similar between them. In their article, Wu et al.^24^ demonstrate the significant role of dedoping anions and the differences between large hydrophobic anions with smaller hydration shells (PF_6_^–^) and smaller hydrophilic anions (Cl^–^). While Cl^–^ is proved to alter the molecular packing of n-PBDF, PF_6_^–^ has fewer water molecules in its hydration shell and can penetrate in the hydrophobic polymer more effectively. This could explain the differences in the *I*_ds_ *–t* profiles observed for the different anions. Here, in order to better understand the role of H^+^ in the long term increase of conductance, we performed experiments using 0.1 M NaCl and HCl electrolytes with different pH. This study was completed using NaCl electrolyte instead of the NaPF_6_ because controlling the pH of the PF_6_^–^-based electrolytes is more complex due to the decomposition of PF_6_^–^, which yields H^+^ altering the pH. NaCl-containing electrolytes at pH of 5.5, 3.5, and 2.0 (Figure S8) show optimum memory response at pH 3.5. We believe that at pH 5.5 the concentration of H^+^ is too low and that at pH 2.0 the strong acidic pH may induce chemical modifications in the polymer. In addition, Figure S9 compares the *I*_ds_ *–t* responses registered in the 0.1 M NaCl (pH 3.5) electrolytes with that obtained for the HCl electrolyte at the same pH. The aim was to remove the Na^+^ to analyze the response of H^+^ exclusively. Interestingly, the HCl electrolytes give rise to a decay in the *I*_ds_ very similar to that of the 0.1 M NaCl electrolytes for the same pH. These observations suggest that the long-term change in conductance is primarily governed by the H^+^. Nevertheless, the presence of Na^+^ does modify the profile of the *I*_ds_ *–t* curve during the *V*_gs_ pulse application, evidencing their role in the n-PBDF doping upon *V*_gs_ bias along with the H^+^. We believe that even though both cations dope the polymer, there must be large differences in the degree of penetration inside the organic layer. Further experiments are planned to understand the penetration depth of the different cations into the polymer layer. These results demonstrate the importance of the electrolyte choice for OECTs and prove that acid electrolytes can trigger the synaptic plasticity of the n-PBDF in OECTs, most likely because of the interaction of H^+^ with the n-PBDF through hydrogen bonds.

Long-term plasticity is achieved through STP under conditions of prolonged training or varying stimulus intensity. It is crucial for transforming short-term memory (volatile) into long-term memory (nonvolatile). To test the performance of the n-PBDF OECT for long-term plasticity, we performed experiments in which consecutive excitatory (writing) and inhibitory (erasing) *V*_gs_ pulses were applied to mimic the synaptic characteristics of long-term potentiation (LTP) and long-term depression (LTD). In these experiments, we first determined the optimal values for several key parameters, such as the setup gate voltage (Figure S10), reset gate voltage (Figure S11), pulse width (Figure S12), and pulse number (Figure S13). The optimal pulse width and pulse number are interrelated; therefore, the optimal pulse number is 50 for a pulse width of 50 ms and 10 for a pulse width of 500 ms. For all the experiments, we held the reading *V*_gs_ at 0 V and the *V*_ds_ at 1.2 V. The results revealed that the optimal LTP/LTD curves (Figure 4a,b) were obtained when *V*_gs_ = 0.8 V and the pulse width is 50 ms (50 pulses) or 500 ms (10 pulses). The device responds to positive voltages with conductance increasing linearly with the number of pulses. By subsequently applying negative pulses, the conductance decreases with the number of pulses until it returns to the device’s initial conductance state. This conclusion was confirmed by simulating a DNN for handwritten digit recognition based on the modified National Institute of Standards and Technology (MNIST) data set, where the weight modulation was conducted based on the behavior of the n-PBDF OECTs. The model comprises an input layer with 784 neurons corresponding to 28 × 28-digit data; two hidden layers with 256 and 64 neurons, respectively; and an output layer with 10 neurons representing digits from 0 to 9. The DNN based on the n-PBDF OECT achieved a recognition rate of 96% (50 ms) and 97% (500 ms), demonstrating the device’s outstanding classification capability (Figure S14). To further explore the potential of the n-PBDF OECT device, we added Gaussian noise at different rates (i.e., 30%, 50%, and 70%) during DNN training to emulate more challenging conditions in practical applications. Figure 4 shows the results of the investigation.

**Figure 4:**
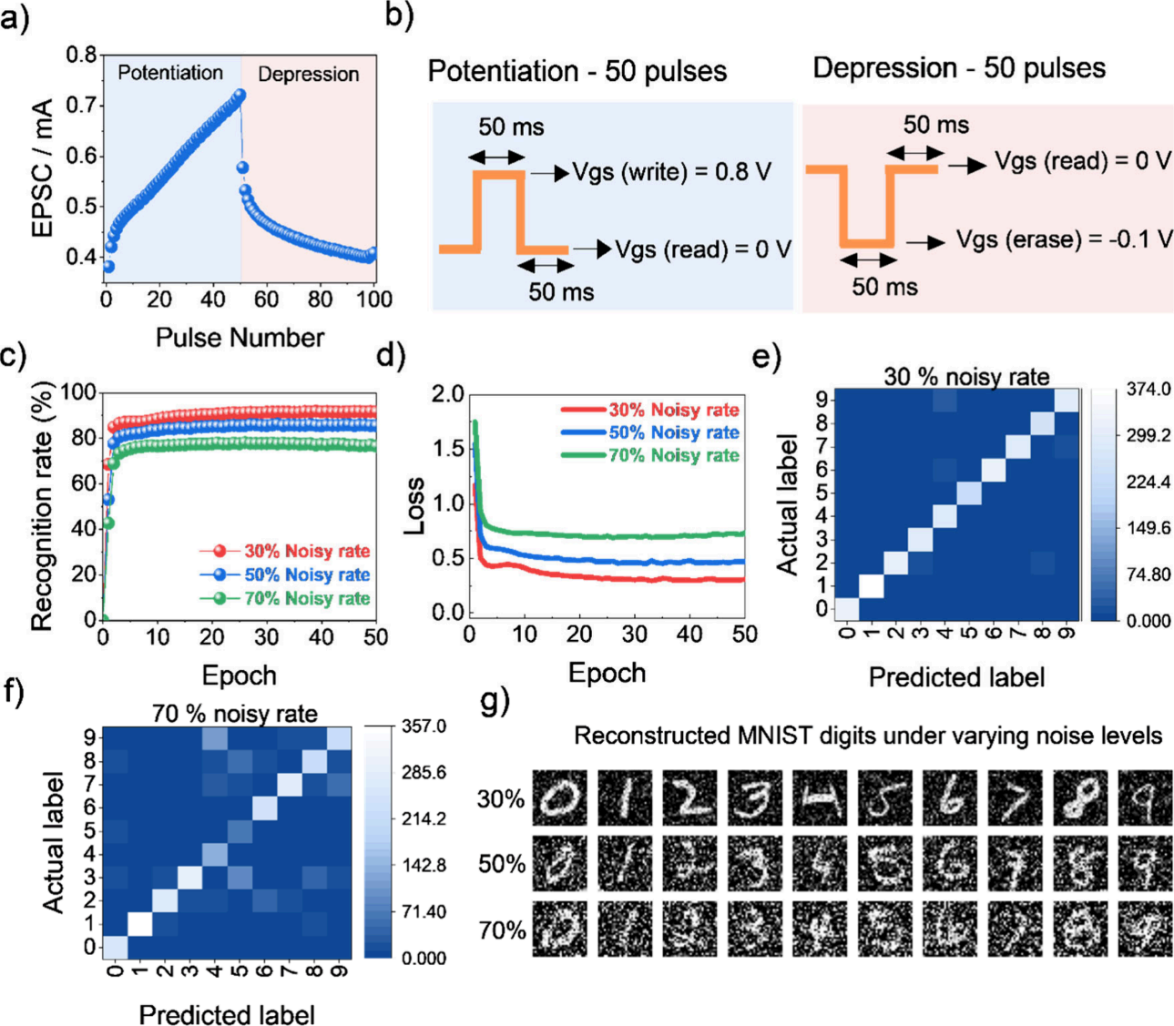
Characteristics of the n-PBDF OECT in actual neuromorphic computing. (a) Long-term potentiation (LTP) and long-term depression (LTD) curves obtained through a sequence of *V*_gs_ pulses. (b) Schematic diagram of excitatory (potentiation) and inhibitory (depression) *V*_gs_ pulse sequences used to construct LTP/LTD curves. (c) Relationship between recognition accuracy and training epoch number of a deep neural network (DNN) trained for handwritten digit classification under various noise input conditions (noise rates of 30%, 50%, and 70%). (d) Loss versus training epoch plot under different noise rates. (e and f) Confusion matrices under 30% and 70% noise rates after training for 50 epochs. (g) DNN-based MNIST handwritten digit reconstruction with different Gaussian noise.

Figure 4a shows the LTP/LTD curves obtained under the conditions described in Figure 4b, proving the successful emulation of synaptic properties of LTP/LTD. Figure 4c,d shows the results of image recognition simulations conducted on the device during training in a DNN at different rates (30, 50, and 70%), with noise present in the input during training. Figure 4c presents the recognition accuracy of the DNN under different Gaussian noise levels. The computing results show successful learning, with accuracy rapidly improving and reaching saturation. The results reveal that the system based on n-PBDF OECT has high fault tolerance, maintaining accuracy above 76% even after 50 training epochs and even under a high Gaussian noise rate of 70%. Figure 4d further confirms the reliability of the device, as the loss converges with increasing training times and shows negligible overfitting, even at a 70% noise rate. Figure 4e,f shows the confusion matrices simulated at 30% and 70% noise levels. In both plots, the heatmaps of actual labels versus predicted labels present a diagonal distribution, demonstrating that the DNN based on n-PBDF OECT correctly classifies previously unseen handwritten digits from 0 to 9. Finally, Figure 4g provides the reconstructed MNIST digits at different noise levels, highlighting the system’s robust information retention and feature extraction capabilities. These results demonstrate the capacity of the n-PBDF OECT to emulate synaptic behavior and its potential in neuromorphic computing applications. The n-PBDF OECT showed suitable LTP/LTD behavior at low operating voltages, performed excellently in digit recognition, and demonstrated resilience under high interferences, suggesting that the system is suitable for real-world, noise-prone environments.

In conclusion, we have demonstrated the potential of the n-PBDF polymer to fabricate high-performance n-type OMIEC-based OECTs suitable for neuromorphic applications. We have proven that the n-PBDF-based OECTs show high stability in NaPF_6_ electrolyte, enable resistive switching to meet memory application requirements, and demonstrate synaptic plasticity. The device responds to gate voltage pulses, with conductance varying with the voltage magnitude and polarity (SVDP), the pulse width (SWDP), the number of applied pulses, and their frequency. We have also proven that H^+^ in the electrolyte plays a key role in the change of conductance of n-PBDF, thereby realizing synaptic plasticity behavior. Upon optimization, the n-PBDF OECT device shows high-quality long-term potentiation (LTP)/long-term depression (LTD) behavior at low voltages (0.8 V) and short pulses (50 −500 ms), which is advantageous for reducing energy consumption. To validate the high performance of this device in neuromorphic computing, we have simulated a deep neural network (DNN) for handwritten digit recognition, with weights modulated based on the behavior of the n-PBDF OECT. The n-PBDF-based DNN achieved over 96% accuracy in recognizing MNIST handwritten digits. By adding Gaussian noise to the signals during training, the capabilities and resilience of the n-PBDF-based DNN were further validated, with accuracy remaining at 76% even at a 70% noise rate. These results provide the first evidence that the recently developed n-type polymer n-PBDF exhibits the record conductivity and stability among n-type polymers, demonstrating great potential for applications in electronic device development. This is also the first demonstration of the dual operating mode of n-PBDF in OECTs, as well as the first example of an n-type OECT capable of operating in dual modes (accumulation and depletion). The work opens new avenues for future research, which will drive the development of OECTs based on n-type OMIECs for building (bio)­electronic circuits with broad application prospects, such as (bio)­sensing, memory, and neuromorphic computing.

## Experimental Section

### Chemicals and Materials

Dimethyl sulfoxide (DMSO, anhydrous, 99.9%) and toluene (anhydrous, 99.8%) are purchased from Sigma-Aldrich. Acetone (>99%) and isopropyl alcohol (>99%) are purchased from VWR CHEMICALS (France). Hydrochloric acid (37%) and NaCl (>99.5%) are purchased from Fischer Scientific. NaPF_6_ (98%) is acquired from Merck. The electrolytes used in the OECTs were always prepared in ultrapure water (Millipore). Interdigitated ITO-prepatterned glass substrates (S161: width × length: 30 mm × 50 μm) were acquired from Ossila, and the ITO electrodes were used as the source and drain.

### Synthesis of the PBFDO or n-PBDF Polymer

The synthesis of the PBFDO or n-PBDF polymer (n-doped benzo­[1,2-*b*-4,5-b′]­difuran-2,6-(3H,7H)-dione polymer) was performed following the method reported by Ke and co-workers (2023).^22^ In brief, the synthesis involves copper-catalyzed oxidative polymerization of monomer BDF (benzo­[1,2-*b*-4,5-b0]­difuran-2,6-(3H,7H)-dione) in air, followed by water-mediated doping. The monomer BDF was also synthesized in-house following the article published in 2017 by Singla et al.^31^ The synthesis of the PBFDO/n-PBDF yields a polymer ink with 15 mg mL^–1^ in DMSO. The polymer is always stored in DMSO, and the as-prepared ink is directly used for device preparation. The full conversion of the monomer BDF into the PBFDO polymer was confirmed by ^1^H NMR (Figure S1).

### Fabrication of the n-PBDF OECTs

The n-PBDF solution was prepared by diluting the polymer ink coming directly from the reaction (15 mg mL^–1^) to 10 mg mL^–1^ using DMSO. The solution was sonicated at room temperature in an ultrasonic bath and stirred with a magnetic stirrer for at least 1 h at 45 °C. After this process, the polymer solution should not contain large solid particles. Next, we added butanol into the polymer solution in a butanol:polymer solution volumetric ratio of 1:2. Then we sonicated the mixture at room temperature in an ultrasonic bath for 2 min to ensure proper mixing. Immediately before deposition, the final polymer solution was filtered with a 45 μm pore size PVDF syringe filter to remove all the solids. Before deposition of the n-PBDF film, the ITO-patterned substrates were thoroughly washed to ensure a clean surface. First, the substrates were sonicated in deionized (DI) water with detergent for 10 min, followed by rinsing three times with DI water. Second, they were sonicated in acetone and isopropyl alcohol (IPA) for 10 min each. Finally, the substrates were dried using nitrogen flow and treated with a UV-ozone lamp for 15 min to remove any residual organics and improve surface wettability. n-PBDF channel layers were deposited by the spin-coating technique in air atmosphere. A volume of 30 μL of the as-prepared n-PBDF solution was cast onto the substrate and spun at a rate of 1000 rpm for 1 min. After the spin-coating, the substrates were dried following two consecutive steps: (1) in a hot plate at 50 °C for 10 min and (2) in a vacuum-oven at room temperature for at least 5 h.

### Electrochemical Measurements with the n-PBDF OECTs

Unless otherwise stated, 0.1 M of sodium hexafluorophosphate (NaPF_6_) aqueous solution was employed as the electrolyte. The as-prepared 0.1 M NaPF_6_ electrolyte is naturally acidic, with a pH of ∼3.5. Experiments using 0.1 M NaCl (pH 3.5, 7) aqueous solutions were also performed for testing the stability and the electrolyte–surface interaction. A concentrated HCl aqueous solution was used to adjust the pH of the NaCl solution to pH 5.5, 3.5, and 2. A Ag wire was employed as the gate electrode. *I*–*V* curves of the OECTs were measured using a two-channel Keithley Source Meter controlled by a customized LabVIEW program. The characteristic transfer curves of the OECTs were registered with gate voltages (*V*_gs_) scanned between −0.5 and 0.5 at 0.03 V s^–1^ and different drain biases (*V*_ds_). During transfer curve measurements, *V*_ds_ was held between 0 and 0.5 V. The output curves of the devices were performed by cycling the *V*_ds_ between 0 and 1.5 at 0.03 V s^–1^ and different *V*_gs_ (from 0.5 to −0.5 V). Each *V*_gs_ value was held constant at a specific value for an entire output curve measurement. The application of *V*_gs_ pulses during the characterization of the devices toward synaptic plasticity capabilities was conducted using an Agilent 2202B Arbitrary Waveform generator controlled by custom Python software. For the pulse experiments, *I*_ds_ was measured using a Metrohm’s Autolab PGSTAT204 coupled with Nova 2.1 software. In the electrochemical experiments of the Supporting Information (Figures S8 and S9) the polymer was purified by dialysis to rule out the influence of Cu cations on the memory effect that could remain from the synthesis.

### UV–vis–NIR Spectra Measurements of the n-PBDF OECTs

The transmittance/absorption spectra of the n-PBDF OECTs were obtained using a UV–vis–NIR spectrophotometer Lambda 1050+ (PerkinElmer). Measurements were carried out on complete OECT devices in the configuration Glass/ITO/Polymer.

### Electrical Conductivity Measurement of the n-PBDF Thin Films

The electrical conductivity of the n-PBDF was measured through thin films deposited on glass substrates using the 4-probe method. First, thin films of the as-prepared n-PBDF were spin-coated on previously washed bare glass substrates using the same conditions described for the fabrication of the OECTs. Next, we deposited Ag fingers by thermal evaporation to be used as electrodes. The evaporation of Ag is carried out under vacuum at 8.5 × 10^–6^ Torr with high control of the evaporation rate. 60 nm of Ag are deposited in three steps: 0–2 nm thick Ag at 0.1 Å s^–1^, 2–10 nm at 0.5 Å s^–1^, and 10–60 nm thick Ag at 1 Å s^–1^. The electrical conductivity is calculated by measuring the *I*–*V* and EIS response of the four-electrode device Ag/n-PBDF/Glass by a Metrohm’s Autolab PGSTAT204 coupled with Nova 2.1 software. The thicknesses of the n-PBDF films were measured by a profilometer.

### Deep Neural Network Model Based on the MNIST Database of Handwritten Digits

The Modified National Institute of Standards and Technology (MNIST) database consisted of 60,000 handwritten digit images, each with a 28 × 28-pixel size. The deep neural network (DNN) architecture is composed of an input layer (784 neurons connected to the image features; two hidden layers with 256 and 128 neurons, respectively; and an output layer (10 neurons that correspond to the handwritten digits). The computing model was conducted with a python-coded program. Additionally, the long-term potentiation/depression (LTP/D) was implemented into the synaptic weights for realizing neuromorphics. Then, the Gaussian noise was carried out at different noise rates (30%, 50%, 70%) to evaluate robustness in this DNN model.

## Supplementary Material



## Notes

^1^H-NMR characterization, electrical conductivity measurements, additional data from the experiments, and simulation of the deep neural network without Gaussian noise (PDF)
