# Supplementary material for: Proton Migration-Modulated n‑Doped Poly(benzodifurandione) Organic Electrochemical Transistors Used for Neuromorphic Computing Applications
Source: ACS Energy Lett. 2025 Oct 2;10(11):5209–17. doi: 10.1021/acsenergylett.5c02076 (PMC12626095; doi:10.1021/acsenergylett.5c02076)
Supplement: Supplementary file 1 [file nz5c02076_si_001.pdf]

Supporting Information

Proton migration-modulated n-doped poly(benzodifurandione) (PBFDO/n-PBDF) organic electrochemical transistors used for neuromorphic computing applications

Ignacio Sanjuán,[a,] David Franco,[a] Qun-Gao Chen,[b] Chu-Chen Chueh,[c] Wen-Ya Lee,[d] Antonio Guerrero[a,]**

[a] Ignacio Sanjuán, David Franco, Antonio Guerrero

Institute of Advanced Materials (INAM), Universitat Jaume I, 12006 Castelló, Spain. *E-mail corresponding authors: isanjuan@uji.es, aguerrer@uji.es

[b] Qun-Gao Chen

Department of Chemical Engineering and Biotechnology, National Taipei University of Technology, Taipei 106344, Taiwan.

[c] Chu-Chen Chueh

Department of Chemical Engineering, National Taiwan University, Taipei 10617, Taiwan.

[d] Wen-Ya Lee

Department of Chemical Engineering and Biotechnology, and High-Value Biomaterials Research and Commercialization Center, National Taipei University of Technology, Taipei 106344, Taiwan.

Keywords: OECTs, PBFDO/n-PBDF, neuromorphic computing, artificial synapsis, n-doped polymers.

H^1-NMR Spectra of monomer and PBFDO/n-PBDF

The H^1-NMR of the pure monomer is described in **Fig. S1a** (top). The peaks at 7.25 and 3.96 ppm correspond to the aromatic hydrogen and the methylene hydrogen, respectively. The synthesis of the monomer is described in the main text and the product is pure at >99 % by H^1-NMR with DMSO-d_6 and residual water as the only other signals observed. **Fig. S1b** (bottom) shows the magnified H^1-NMR spectrum of the polymer. As reported previously, the signals of the polymer are not observed using this analytical technique due to the doping of the polymer. At high magnifications it is possible to observe residual signals and exchange signals of H^+ with the residual water. The complete monomer conversion is justified by the disappearance of the BDF peaks in the PBFDO/n-PBDF spectrum.

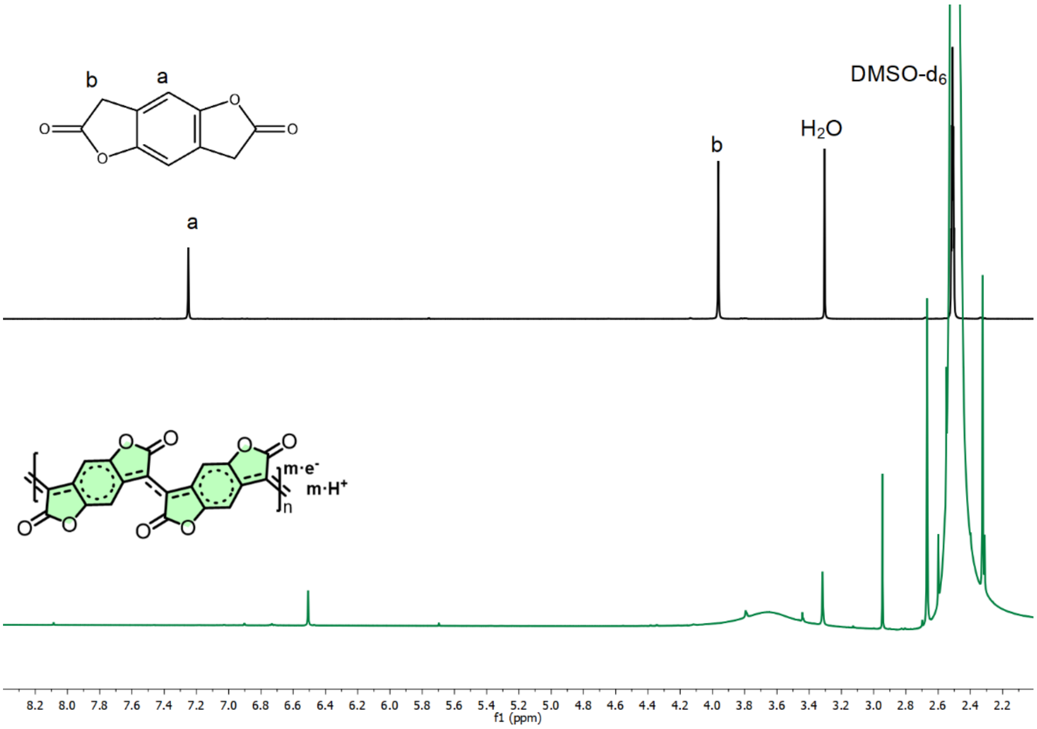

Figure S1. NMR spectra of the monomer BDF (top) and the as prepared polymer PBFDO/n-PBDF (bottom) in DMSO-d_6.

Electrical conductivity measurements of PBFDO/n-PBDF

A typical 4-probe set up is used to measure the conductivity and the results are corroborated by using impedance spectroscopy.

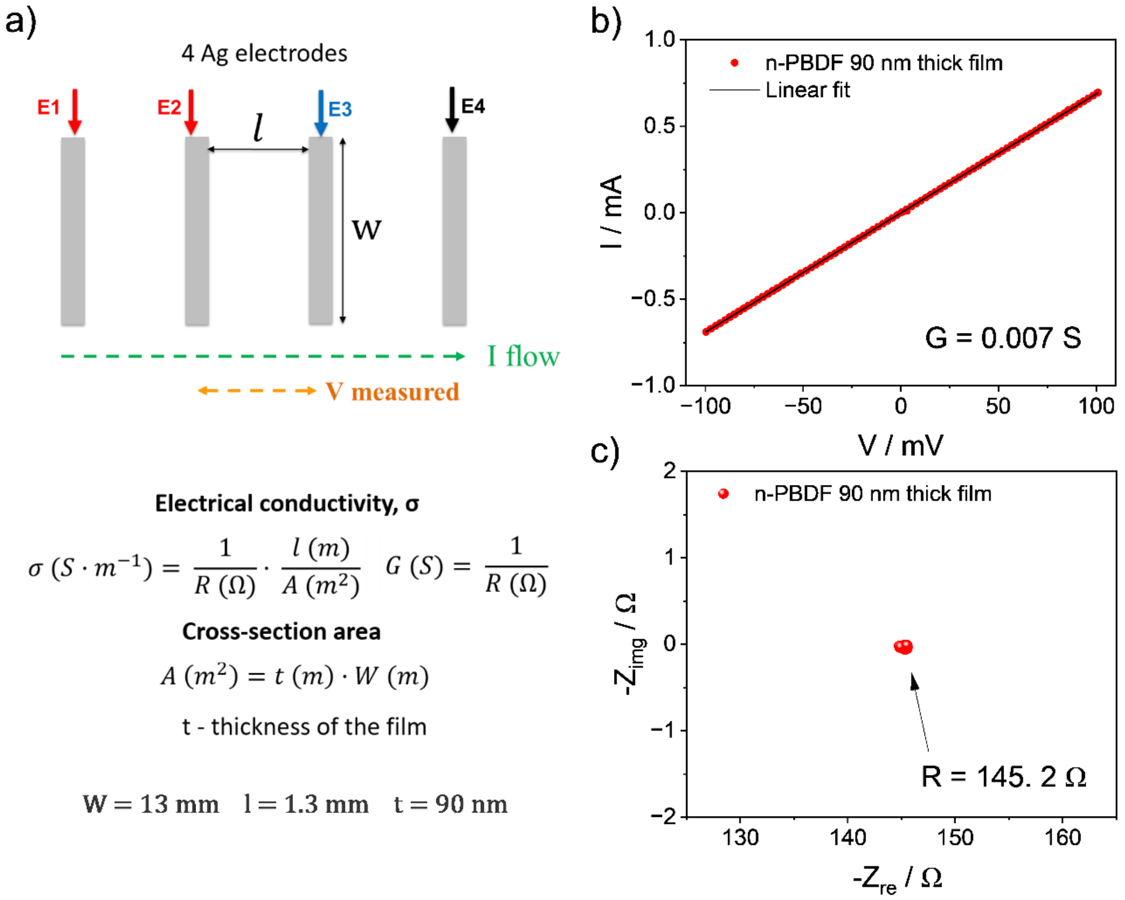

Figure S2. Electrical conductivity measurements of the PBFDO/n-PBDF film. a) Scheme illustrating the principle of the 4-probe method used for the electrical conductivity measurement and the equations. b) Representative I-V voltammetry plot used for the measurement of the conductance. c) Representative Nyquist Plot used for the analysis of the film impedance.

Cyclic voltammetry stability measurements using NaCl and $NaPF_6$

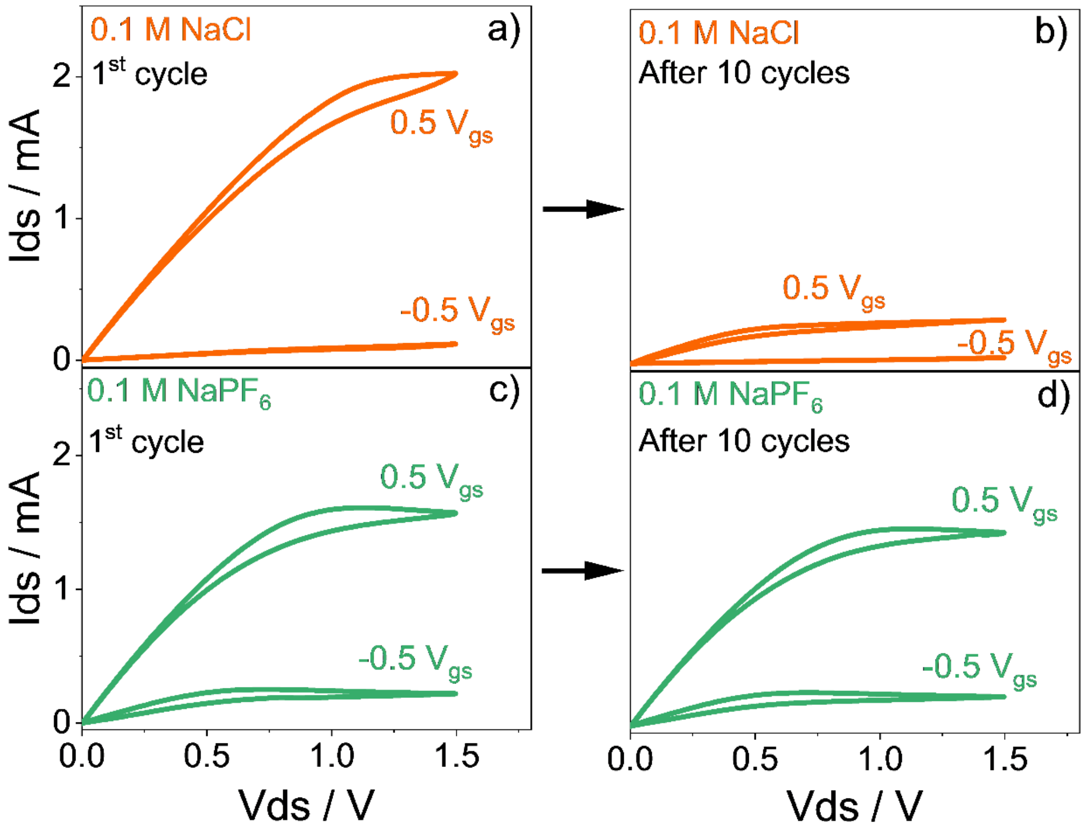

Figure S3. Consecutive cycles of output curves registered in different electrolytes with the n-PBDF OECTs**.** Each cycle includes 5 output curves with different V_{gs} (0.5, 0.25, 0, -0.25, 0.5 V) but only 2 are shown. V_{ds} is cycled between 0 and 1.5 V. a) and b) 1st and 10th cycle of output curves measured in 0.1 M NaCl. c) and d) 1st and 10th cycles of output curves measured in 0.1 M $NaPF_6$.

Transconductance plot of devices fabricated with PBFDO/n-PBDF

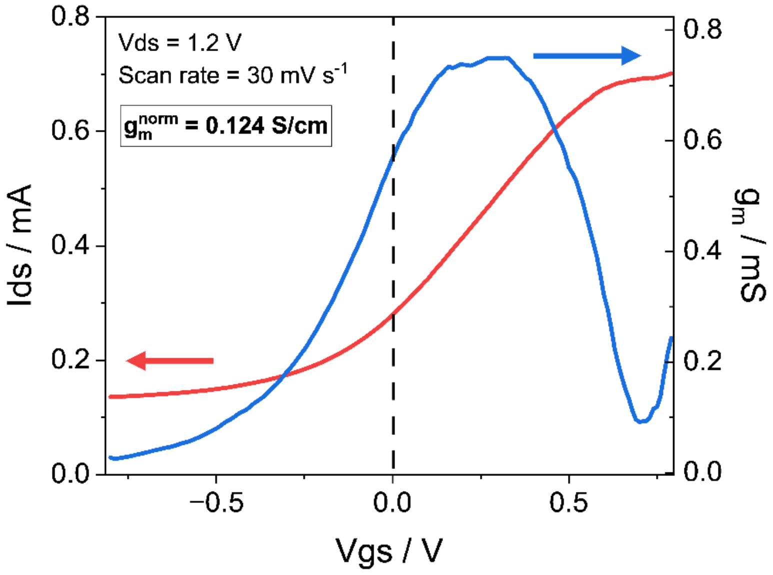

Figure S4. Transfer curve (red) and transconductance (g_m) versus V_{gs} plot for the n-PBDF OECT in 0.1 M $NaPF_6$ (V_{ds} = 1.2, scan rate 30 mV s^{-1}).

Synaptic characterization of devices fabricated with PBFDO/n-PBDF

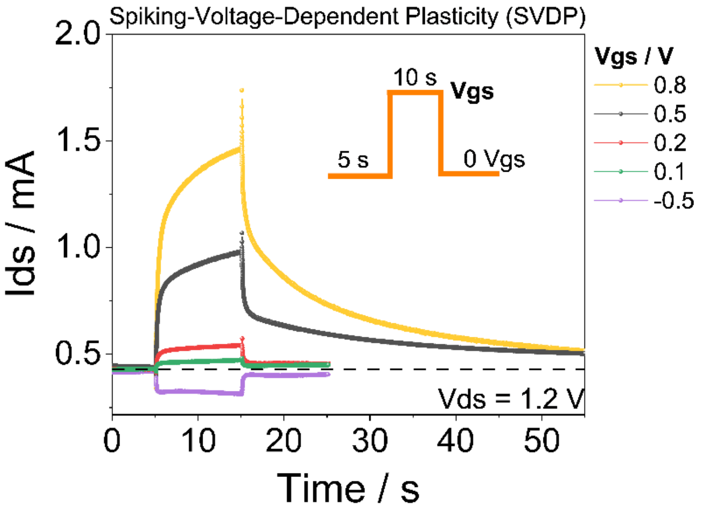

Figure S5. Plot of I_{ds} versus time during the application of a 10-second V_{gs} pulse and 40 s of rest time. Pulses with different V_{gs} between 0.8 and -0.5 V are applied. V_{ds} is held at 1.2 V. The measurements were stopped when the I_{ds} reached a steady value.

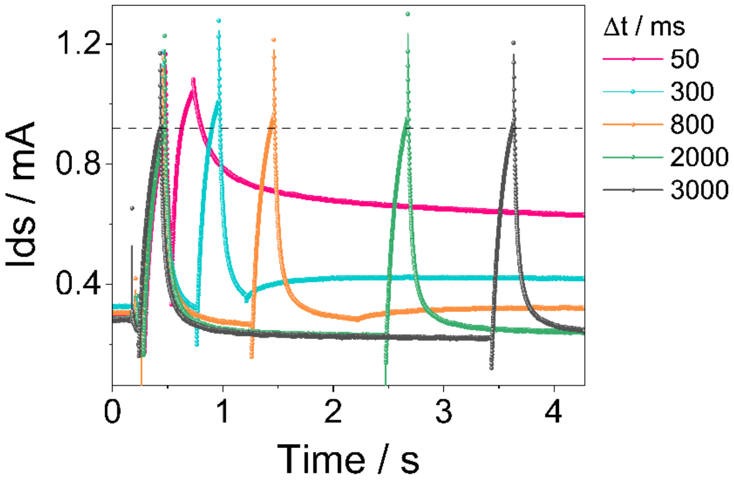

Figure S6. Plot of I_{ds} as a function of time during the application of paired V_{gs} pulses with varying interval times (Δt, the time between pulses). V_{gs} is 0.8 V during each pulse (t_{pulse} = 200 ms*)* and 0 V at the rest time. V_{ds} is held at 1.2 V. The black dashed line indicates the I_{ds} level of the first pulses.

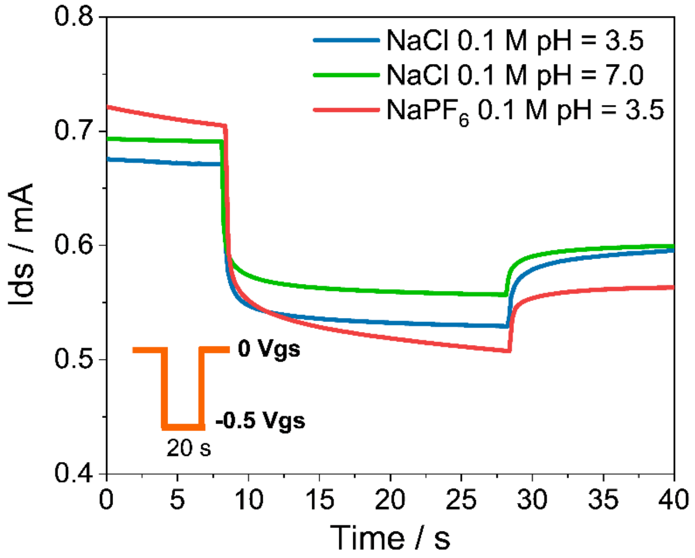

Figure S7. I_{ds} current-time response under a gate voltage V_{gs} pulse [-0.5 V, 20 s] for the n-PBDF OECTs with different electrolytes.

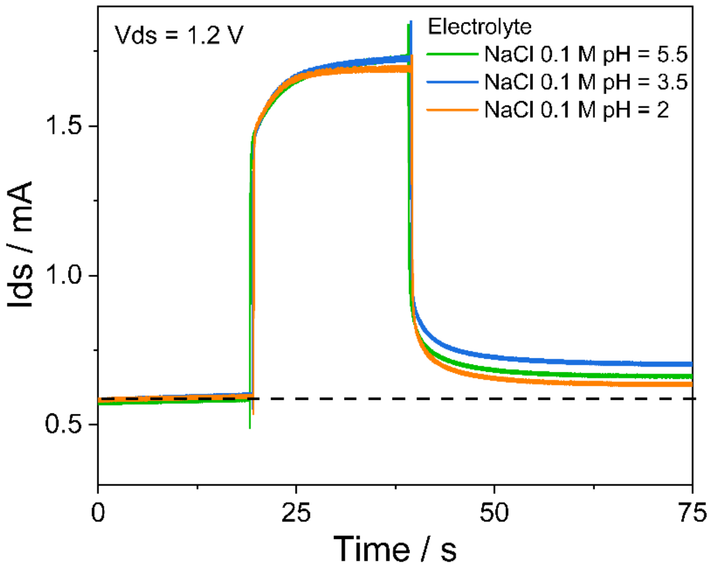

Figure S8. I_{ds} current-time response under a gate voltage V_{gs} pulse [+0.5 V, 20 s] for the n-PBDF OECTs with 0.1 M NaCl electrolytes at different pH values: 5.5, 3.5, and 2.

Figure S8 compares the $I_{ds} - t$ profile of the n-PBDF OECTs in 0.1 M NaCl-containing electrolytes at different pH values, i.e., 5.5, 3.5, and 2. As can be observed, the presence of H^+ always gives rise to a long-term increase of conductance, unlike neutral conditions, and its magnitude depends on the pH. The 0.1 M NaCl (pH = 3.5) electrolyte shows an almost double increase in I_{ds} (~120 µA) than 0.1 M NaCl at pH = 5.5 and 2, suggesting that the pH is a parameter to optimize. A pH of 5.5 would entail a lack of H^+ while highly acidic conditions could promote chemical changes in the polymer structure (e.g., opening of the lactone ring), which has been proved to influence its doping capacity.

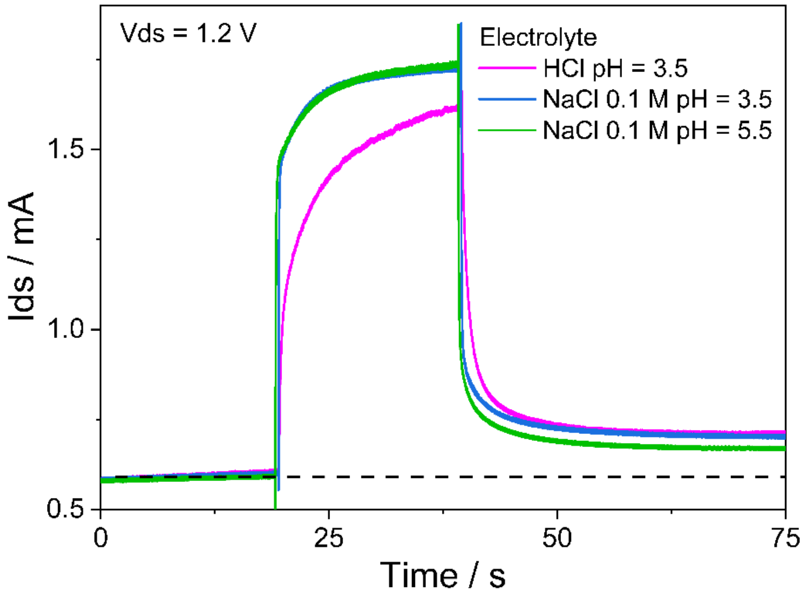

Figure S9. I_{ds} current-time response under a gate voltage V_{gs} pulse [+0.5 V, 20 s] for the n-PBDF OECTs with different electrolytes: HCl pH 3.5, NaCl 0.1 M pH = 3.5 and NaCl 0.1 M pH = 5.5.

Figure S9 reveals that the change in conductance that remains after the *Vgs* pulse depends on the pH, as the responses in NaCl and HCl match for the same pH values.

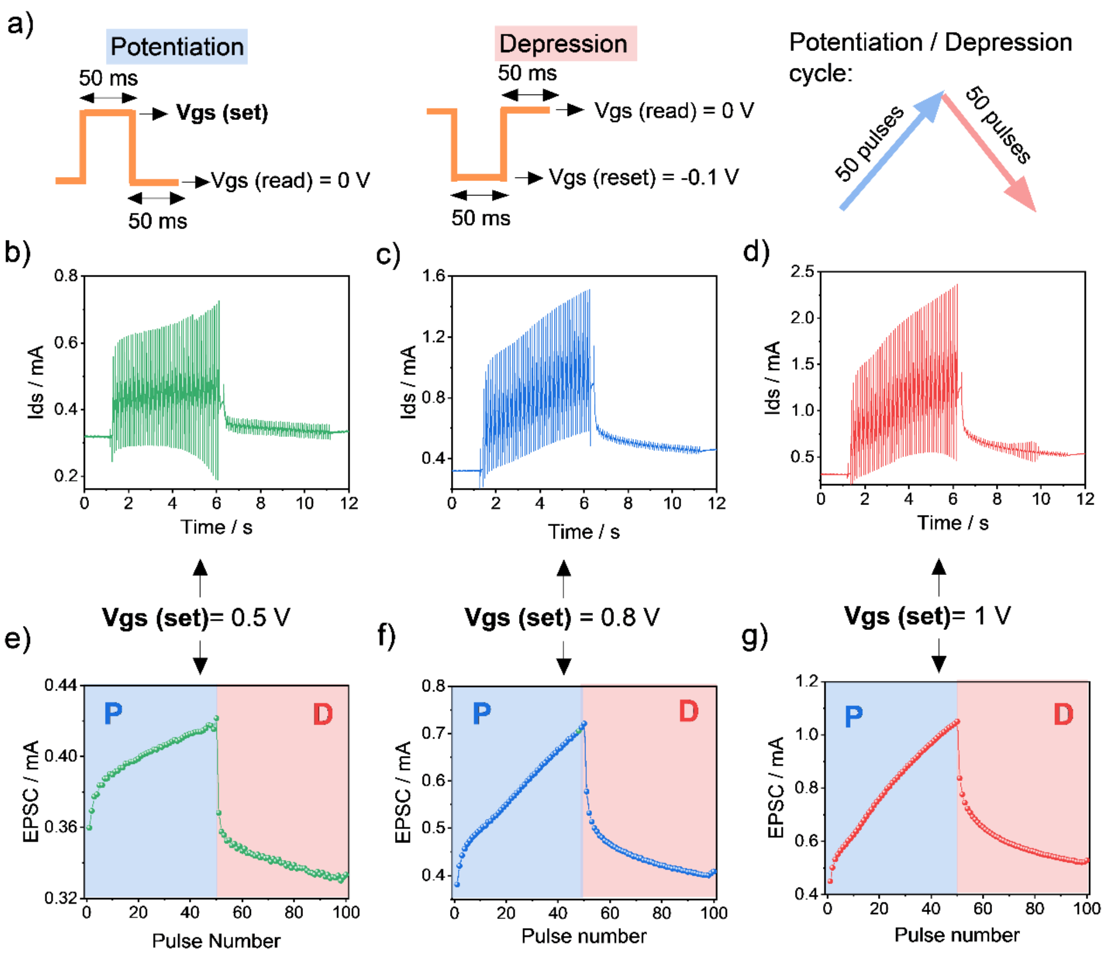

Figure S10. Analysis of the long-term potentiation (LTP) and depression (LTD) behavior of the n-PBDF OECTs upon the application of different gate voltage pulses (V_{gs}). a) Scheme of the experimental program describing the application of sequences of gate voltage pulses for potentiation and depression. b-d) Plot of drain-source current (I_{ds}) versus time registered under the application of the LTP/LTD gate voltage sequences with different V_{gs} *(set)* = 0.5, 0.8, and 1 V, respectively. e-g) LTP/LTD cycles of excitatory post-synaptic currents (EPSC) with the pulse number applied, derived from the data of (b-d).

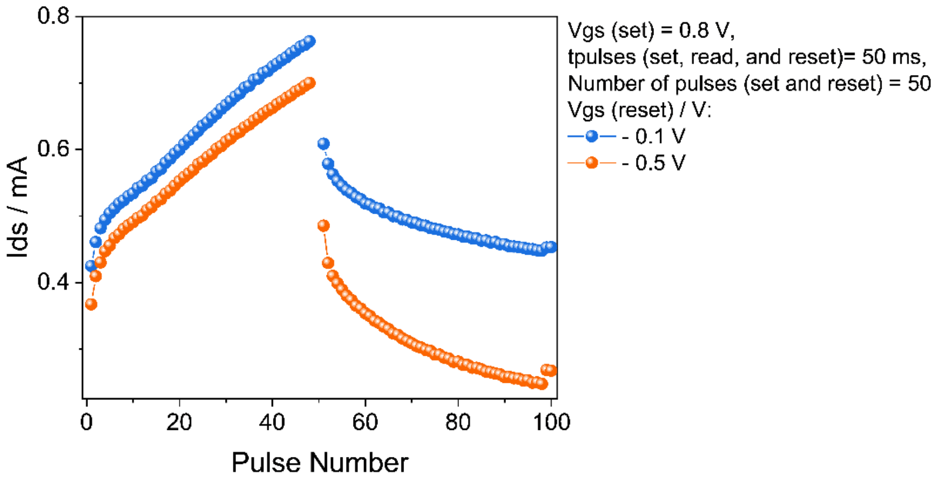

Figure S11. Long-term potentiation (LTP) and depression (LTD) curve obtained for a n-PBDF OECT device with different V_{gs} (reset) voltages for depression and maintaining the same values for the rest of operating parameters, as described in the legend.

Figure S11 reveals that slightly negative V_{gs} are optimal for the LTP/LTD behavior when V_{gs} (set) = 0.8 V since the use of more negative V_{gs} leads to a device conductance level that is lower than the initial state of the device before potentiation.

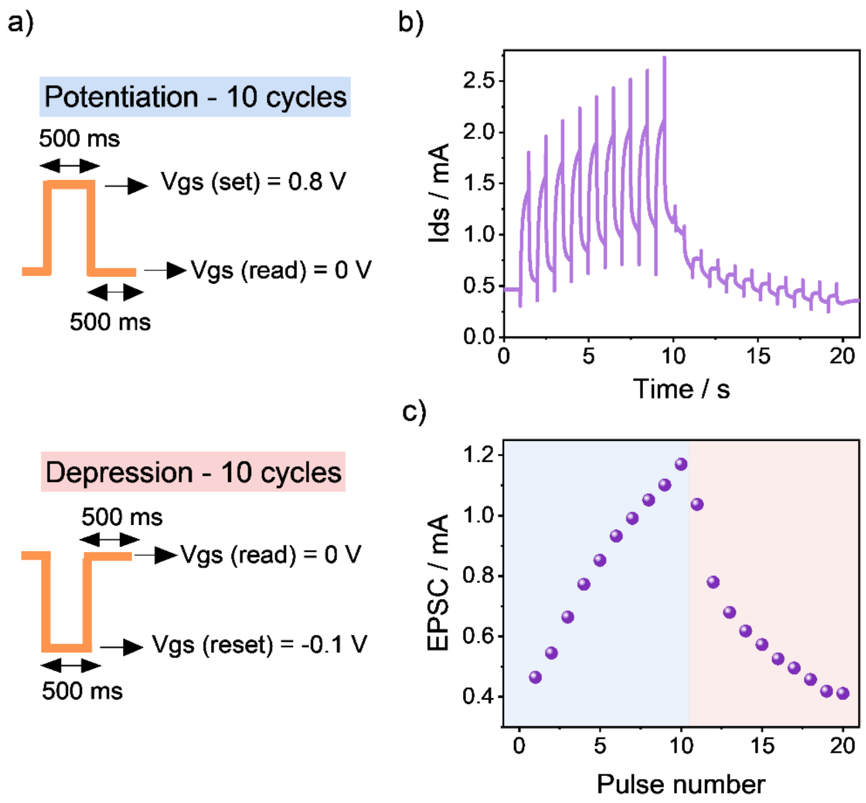

Figure S12. Analysis of the long-term potentiation (LTP) and depression (LTD) behavior of the n-PBDF OECTs with longer pulse width (500 ms) and lower number of pulses. a) Scheme of the experimental program describing the application of sequences of gate voltage pulses for potentiation and depression. b) Plot of drain-source current (I_{ds}) versus time registered under the application of the LTP/LTD gate voltage sequences with V_{gs} *(set)* = 0.8 V. c) LTP/LTD cycles of excitatory post-synaptic currents (EPSC) with the pulse number applied, derived from the data of (b).

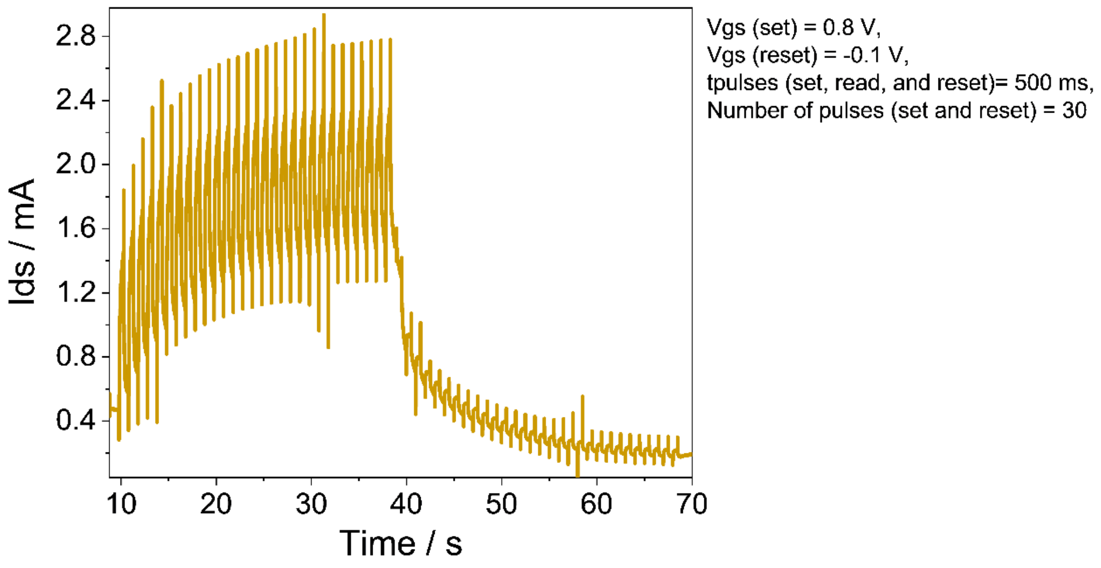

Figure S13. Plot of drain-source current (I_{ds}) versus time registered under the application of 500 ms-long LTP/LTD gate voltage sequences. 30 pulses for potentiation (LTP) and 30 pulses for depression (LTD).

Figure S13 illustrates the importance of the pulse number used for performing the LTP/LTD cycles. In this experiment, long pulses were used (500 ms) and therefore 30 pulses were saturating the conductance of the n-PBDF OECT device during LTP with no significant raise in the drain-source current approximately from the cycle number 20. This effect gives rise to non-linear LTP curves, which is not beneficial for the practical application. In these conditions, we considered 10 cycles as the optimal pulse number for LTP and LTD aiming at a linear LTP response. When the V_{gs} pulses are shorter (Figure S10c and f), the LTP curve maintain the linear increase in the current up to 50 cycles at least.

Characterization of the n-PBDF OECTs for handwritten digit recognition using the long-term potentiation/depression

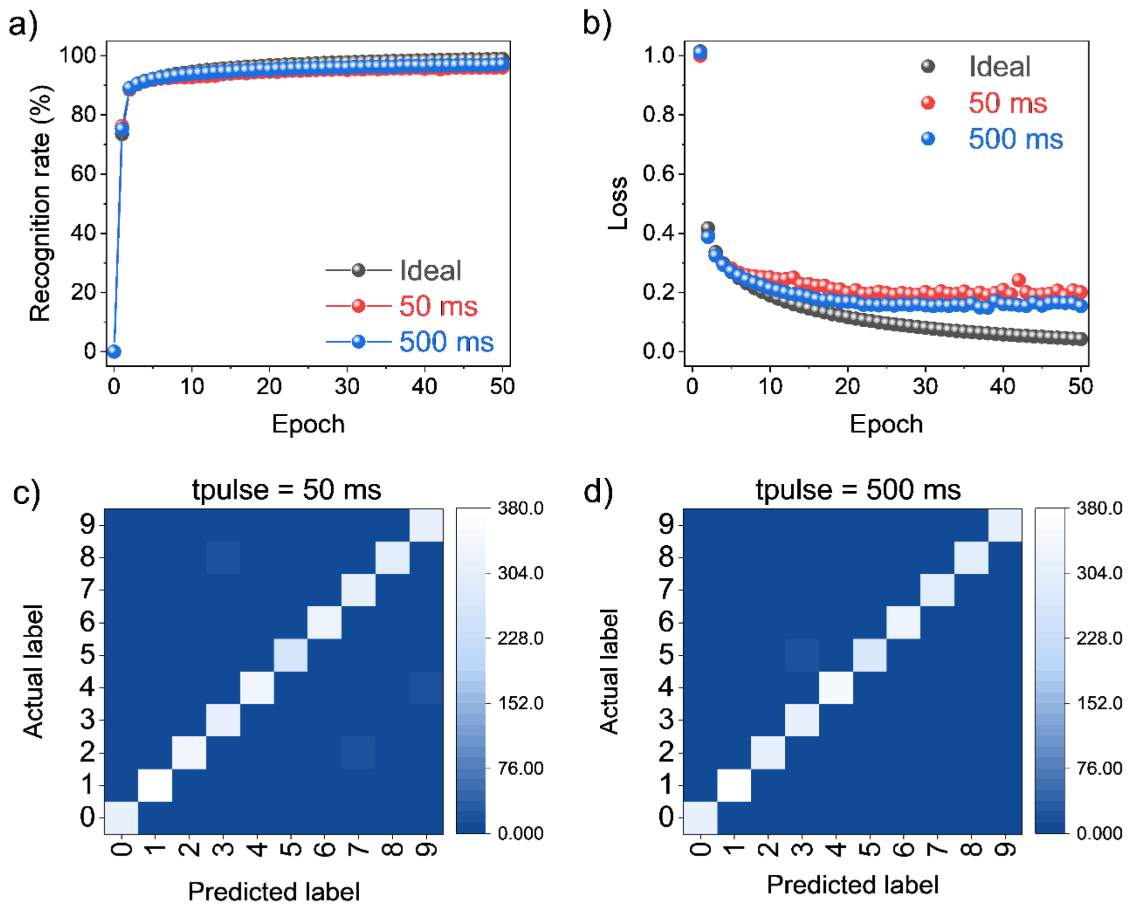

Figure S14. Characterization of the n-PBDF OECTs for handwritten digit recognition using the long-term potentiation/depression (LTP/LTD) behavior in the optimal conditions: V_{gs} (set) = 0.8 V, V_{gs} (reset) = -0.1 V, t_{pulse} (set/reset) = 50 ms [50 pulses]/500 ms [10 pulses]. (a) Plot of recognition accuracy with the number of epochs of a deep neural network DNN trained for handwritten digit classification, comparing the accuracy of the n-PBDF OECT device with the ideal case. (b) Loss versus training epochs plot for the different LTP/LTD conditions and their comparison with the ideal case. (c-d) Confusion matrices obtained after training 50 epochs using the features of the n-PBDF OECTs under different conditions (pulse number and pulse width).
